# A Preliminary Study on the Relationship between Parasitaemia and Cytokine Expression of Peripheral Blood Cells in *Trypanosoma vivax*-Experimentally Infected Cattle

**DOI:** 10.3390/ani11113191

**Published:** 2021-11-08

**Authors:** Otavio Luiz Fidelis Junior, Paulo Henrique Sampaio, Luiz Ricardo Gonçalves, Rosangela Zacarias Machado, Marcos Rogério André, Gene Wijffels, Fabiano Antonio Cadioli

**Affiliations:** 1 Department of Veterinary Medicine, Vila Velha University (UVV), Avenida Comissário José Dantas de Melo 21, Vila Velha 29102-920, ES, Brazil; otaluf@gmail.com; 2 Laboratory of Immunoparasitology, Departament of Pathology, Reproduction and One Health, School of Agricultural and Veterinarian Sciences, São Paulo State University (Unesp), Via de Acesso Paulo Donato Castellane s/n, Jaboticabal 14884-900, SP, Brazil; paulohsampa@yahoo.com.br (P.H.S.); luizbio08@outlook.com (L.R.G.); rzacariasmachado@gmail.com (R.Z.M.); mr.andre@unesp.br (M.R.A.); 3 Commonwealth Scientific and Industrial Research Organization (CSIRO), 306 Carmody Rd, St. Lucia, QLD 4067, Australia; gene.wijffels@csiro.au; 4 School of Veterinary Medicine, São Paulo State University (Unesp), Araçatuba, Rua Clóvis Pestana 793, Araçatuba 16050-470, SP, Brazil

**Keywords:** trypanosomosis, host–parasite relationship, RT-qPCR, Th1, Th2

## Abstract

**Simple Summary:**

Infections by *Trypanosoma vivax* in livestock have been reported with increasing frequency worldwide. Nevertheless, information regarding the immune response during the infection is scarce. Regarding that, cytokines play an important role as inflammation modulators, influencing the outcome of trypanosomosis. This study aimed to evaluate host cytokine production during *T. vivax* infection, in order to assess the increase or decrease of selected cytokines with the cattle’s ability to control the infection. While animals that showed an increase in IL-6 and IFNγ managed *T. vivax* parasitaemia satisfactorily, cattle that showed reduction of IL-1β, IL-2 and TNFα did not control the parasite multiplication. The presented results are preliminary and shed some light on the role of cytokines during *T. vivax*-infection.

**Abstract:**

*Trypanosoma vivax* outbreaks have been reported with increasing frequency worldwide, causing significant economic losses in livestock. Though several studies have suggested that cytokine responses may influence infection caused by *Trypanosoma* sp., their exact role remains unclear and may vary according to the animal species and parasite strain. The present study aimed to evaluate cytokine expression of peripheral blood cells from three Girolando dairy cows experimentally infected with *T. vivax*. For this purpose, blood samples were collected prior to the inoculation on the day of inoculation (D0), the day after inoculation (D1), and then every seven days up to 119 days after infection (DAI). Each animal presented a unique pattern of cytokine expression. While a tendency of a Th1 cytokine response was observed during the patent phase (presence of circulating parasites), an increase of Th2 cytokine expression was found at the beginning of the sub-patent phase (low parasitaemia or aparasitaemic periods). In animals that presented a better control of parasitaemia, IL-6 and IFNγ increased during most of the trial period. On the other hand, the cow that presented reduction of IL-1β, IL-2, and TNFα during the entire period did not control parasitaemia properly. A balance between the Th1 and Th2 profile is beneficial for parasite control and animal health. The results found in the present study are a first step towards elucidating the dynamics of cattle’s inflammatory response against *T. vivax*, requiring future studies focusing on the role of key cytokines on the controlling of parasitaemia in different stages of bovine trypanosomosis.

## 1. Introduction

Trypanosomosis is a cosmopolitan disease that affects humans and animals. *Trypanosoma brucei*, *T. congolense*, *T. vivax*, and *T. evansi* cause significant economic losses in livestock in Africa, Asia, and Central and South America [1,2]. In South America, the most critical trypanosome species are *T. cruzi*, the agent of Chagas’ disease in humans and dogs, *T. evansi*, that causes neurological disorders in horses and other mammals, and *T. vivax*, which causes productive and reproductive losses in ruminants [3,4,5].

Infections by *T. vivax* in livestock have been reported with increasing frequency worldwide [2,6,7,8]. Fluctuations in parasitaemia and even apparently aparasitaemic intervals have been detected during infection [9,10,11], which may be related to the host’s immune response and the antigenic variation of the variant surface glycoproteins (VSG), which are responsible for stimulating the host to produce cytokines [12,13].

Cytokines are critical modulators of inflammation, participating in both acute and chronic phases of diseases. A better understanding of the regulation mechanisms of these pathways would facilitate a more accurate identification of agents mediating inflammation and, as a consequence, provide more effective treatments [14]. Nevertheless, information regarding cytokine responses during *T. vivax* infection is very limited in the literature. Even though several studies have suggested that cytokine responses may influence infection caused by *Trypanosoma* sp., their exact role remains unclear and may vary according to the animal species, parasite strain, and load [15,16,17]. Thus, the present study aimed to evaluate the relationship between the parasitaemia and cytokine expression of peripheral blood cells during the course of infection by *T. vivax* in experimentally infected cattle, aiming at assessing the host’s innate immune system response pattern in both early and late stages of the disease.

## 2. Materials and Methods

### 2.1. Experimental Infection and Sample Collection

Three Girolando cows, healthy and disease-free, aged six to seven years [18] were experimentally infected with 2.0 × 10^7^ trypomastigotes of *T. vivax* “Lins” isolate [7,19], by intravenous route. Blood sampling of each animal was performed on the day of inoculation (0 DAI), the day after inoculation (1 DAI), and then weekly until 119 days after infection (119 DAI). At each blood collection, 4 mL of whole blood was obtained by jugular venipuncture and collected into a vacutainer tube containing 10% K2-EDTA (B.D., Juiz de Fora, Minas Gerais, Brazil). Two millilitres of whole blood were split into triplicates. The remaining blood (2 mL) was divided into triplicates in DNAse/RNAse-free microtubes containing RNAlater^®^ solution (Life Technologies^®^, Carlsbad, CA, USA), according to the manufacturer’s recommendations. All samples were stored at −80 °C until required for analyses.

### 2.2. qPCR (Quantitative Real-Time PCR) for Trypanosoma vivax Detection and Quantification

The DNA extraction was performed with the QIAamp DNAeasyKit (Qiagen^®^, Germantown, MD, USA) according to the manufacturer’s recommendations, using an aliquot of 200 μL of each EDTA-blood sample. DNA concentration and quality were evaluated in NanoDrop^®^ 2000 (Thermo Scientific^®^, Wilmington, DE, USA), according to the manufacturer’s instructions. The extracted DNA was stored at −20 °C until required.

TaqMan qPCR was performed as described by Silbermayr et al. [20]. Primers for concurrent detection of the ITS1 regions of *T. congolense*, *T. brucei,* and *T. vivax* and for the bovine toll-like-receptor 8 (TLR-8) (endogenous gene) were initially used with the addition of probes labelled with FAM fluorophore and BHQ-2 quencher for *T. vivax* detection and HEX fluorophore and BHQ-2 quencher for TLR-8 detection. Reactions were performed using 5 μL of genomic DNA (except for the negative control), 200 nM of Tryps_KS-for (5′-CGTGTCGCGATGGATGACTT-3′), Tryps_KS-rev (5′- CAAACGGCGCATGGGAG-3′), TLR8-for (5′- TGTTTAGAGGAAAGGGATTGGG-3′) and TLR8-rev (5′-TTGGTTGATGCTCTGCATGAG-3′) primers, 160 nM of *T. vivax* (FAM–ATGACCTGCAGAACCACTCGATTACCCAGT–BHQ2) probe, 120 nM of TLR-8 (HEX–CCCGGGTCTAGCCATCATCGACAA–BHQ2) probe, buffer 2X (6 nM of MgCl_2_, 0.8 mM of dNTPs and 1 U of Taq DNA polymerase, GoTaq Hot Start Polymerase (Promega^®^, Madison, WI, USA). The final volume of each reaction was 25 μL. qPCR amplifications were conducted in low-profile 96-well unskirted PCR plates (Bio-Rad^®^, Hercules, CA, USA) using a CFX96 thermal cycler (Bio-Rad^®^, Hercules, CA, USA) under specific conditions: (1) initial denaturation at 95 °C for 10 min; (2) 45 cycles at 95 °C for 30 s and 61 °C for 1 min; and (3) termination at 72 °C for 1 min. All samples were processed in duplicate.

The sensitivity of the qPCR assay was tested with gBlock^®^ Gene fragments (Integrated DNA Technologies^®^, Coralville, IO, USA) containing the target sequences for amplification of *T. vivax* ITS1 region. Serial dilutions were made in order to construct standards with different concentrations of gBlock^®^ containing the target sequence (2.0 × 10^7^ to 2.0 copies/µL), which were also used as positive controls. The copy number was determined according to the formula (X g/µL DNA/ [gBlock^®^ size (bp) × 660]) × 6.022 × 10^23^ × copies of gBlock^®^/µL). The amplification efficiency (E) was calculated according to the slope of the standard curve of each run according to the following formula (E = 10^–1/slope^). Sterile ultrafiltered water free of DNAse and RNAse (Promega^®^, Madison, WI, USA) was used as a non-template control. The reactions carried out followed the standards established by MIQE (Minimum Information for publication of Quantitative real-time PCR Experiments) [21].

### 2.3. RNA Extraction and cDNA Transcription

RNA extraction was performed using the RiboPure™-Blood Kit (Life Technologies^®^, Carlsbad, CA, USA). Extracted RNA samples were submitted to specific DNAse treatment, in order to eliminate all genomic DNA. All steps were conducted according to the manufacturer’s recommendations. RNA concentration was evaluated in NanoDrop^®^ 2000 (Thermo Scientific^®^, Carlsbad, CA, USA) and RNA quality assessed using the Agilent 2100 Bioanalyzer equipment with the Micro LabChip Kit (Agilent Technologies^®^, Palo Alto, CA USA), both according to the manufacturer’s instructions. The RNA concentration was expressed in ng/μL and the quality by the number of RNA integrity (RIN). An aliquot of the total RNA (approximately 300 ng) was converted into cDNA using GoScript™ Reverse Transcription System (Promega^®^, Madison, WI, USA), according to the manufacturer’s methodology. cDNA samples were stored at −20 °C until required for analyses.

### 2.4. Cytokine Assessment by RT-qPCR

The expression of interleukin-1 beta (IL-1β), IL-2, IL-4, IL-6, IL-10, IL-12 p40, Tumor Necrosis Factor-alpha (TNFα) and Interferon-gamma (IFNγ) and the expression of Glyceraldehyde 3-phosphate dehydrogenase (*gapdh*), Beta-actin (β-actin), Tyrosine 3-mono-oxygenase/tryptophan 5-mono-oxygenase activation protein zeta (YWHAZ) and H3 histone, family 3A (H3F3A) (reference genes) were evaluated. Raw Cq means were imported to the RefFinder online tool (https://www.heartcure.com.au/reffinder/, accessed on 17 May 2017) to evaluate the reference gene stability. RT-qPCR was performed as described by Konnai et al. [22] and Puech et al. [23] using the SYBR Green system. Primers sequences are presented in Table 1. All reactions were performed in duplicate, with sterile ultrafiltered water free of DNAse and RNAse (Promega^®^, Madison, WI, USA) as non-template control and gBlock^®^ Gene fragments (Integrated DNA Technologies^®^, Coralville, IO, USA) as positive controls. RT-qPCR amplifications were conducted in low-profile 96-well unskirted PCR plates (Bio-Rad^®^, Hercules, CA, USA) using a CFX96 thermal cycler (Bio-Rad^®^, Hercules, CA, USA). Melting curves were performed in order to check the specificity of amplicons. For this purpose, after amplification, the temperature was raised from 63 to 96 °C with increments of 0.5 °C every 5 s. The results were read through observation of amplification curves using the Bio-Rad C.F.X. Manager software (Bio-Rad^®^, Hercules, CA, USA), in which the cycle threshold (Cq) of each sample was annotated. The equation proposed by Livack and Schmittgen [24] was used for relative quantification. The sensitivity of the RT-qPCR assay was tested, as described for *T. vivax* qPCR.

## 3. Results

### 3.1. qPCR for Trypanosoma vivax

**Figure 1:**
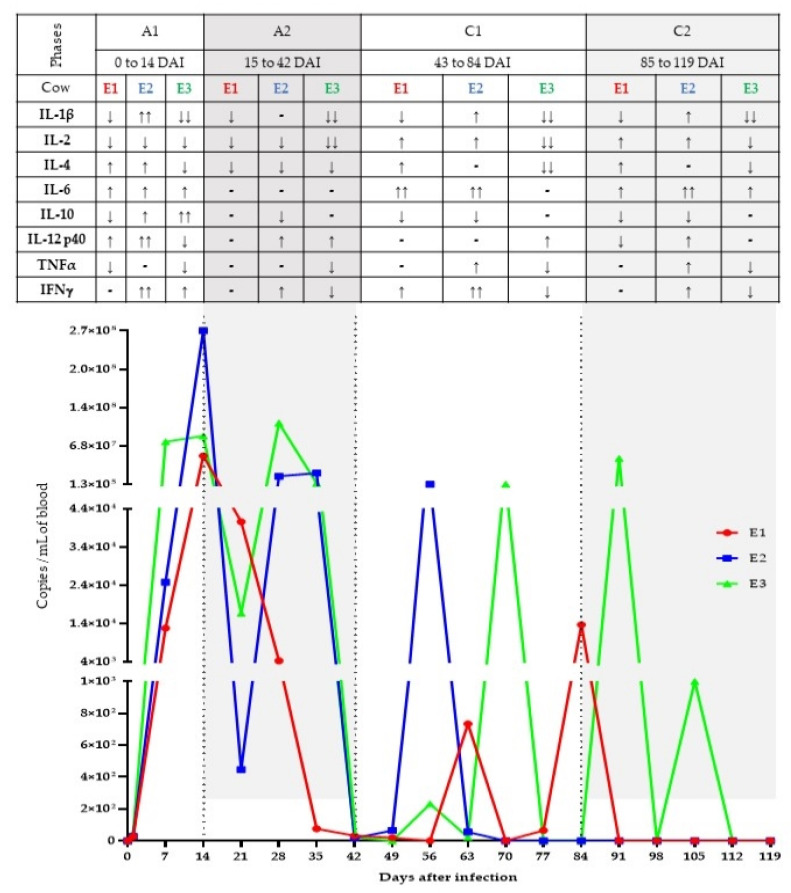
Parasitaemic curves quantified by qPCR (graphic) and cytokines profile (table) of three individual animals (E1, E2, and E3) experimentally infected by *T. vivax*: A1—Early patent phase, when an increase in parasitaemia was found (until an apparent peak); A2—Late patent phase, when a decrease of parasitaemia was found (apparent clearance of parasitaemia); C1—Early sub-patent phase, fluctuations/transient surges in parasitaemia; C2—Late sub-patent phase, absence of *T. vivax* DNA (except for animal E3 which did not control the infection). ↑ - increased; ↑↑ - huge increase; ↓ - decreased; ↓↓ - huge decrease.

The mean and standard derivation of the DNA concentrations and 260/280 and 260/230 ratios obtained from 57 whole-blood samples were 31.6 ± 11.9 ng/μL, 1.9 ± 0.1 and 1.3 ± 0.4, respectively. All samples were positive for the host endogenous gene TLR-8, indicating that extractions were efficient, and giving reliability to the results obtained by qPCR. The mean and intervals for efficiency, R^2^, slope, and y-intercept of qPCR reactions were 93.6%, (90.1–97.1), 0.988 (0.970–0.999), −3.487 (–3.586–(−)3.392) and 39.758 (38.583–41.764), respectively. All duplicates presented a Cq maximum variation of 0.5. *T. vivax* DNA was detected in 33 out of 54 known positive samples (61.1%), with the first detection on the first day after infection (1 DAI). The quantification by qPCR revealed that the highest parasite values occurred on 14 DAI, showing 5.03 × 10^7^, 2.70 × 10^8^ and 8.53 × 10^7^ copies of the target region/mL of blood, for E1, E2, and E3 animals, respectively. Fluctuations in parasitaemia were observed during the experimental period, highlighting a patent phase, in which circulating parasites were seen throughout the entire period, and a sub-patent phase, in which a low parasitaemia was detected followed by aparasitaemic periods. The parasitaemic curves quantified by the qPCR are shown in Figure 1.

### 3.2. Cytokine Assessment by RT-qPCR

The mean and standard derivation of the RNA concentrations, 260/280 and 260/230 ratios and RIN obtained from 57 whole-blood samples maintained in RNAlater^®^ solution were 33.2 ± 19.2 ng/μL, 2.0 ± 0.1, 1.2 ± 0.4 and 8.8 ± 0.4, respectively. Three of the fifty-seven samples had no RIN value (samples from 21 DAI), indicating that the material was possibly degraded. These samples were extracted again, and once more, no RIN values were obtained. These three samples were eliminated from the experiment, and the remaining 54 samples were reverse transcribed into cDNA.

The reaction efficiency and sample Cq variation criteria established by Bustin et al. [21] were employed to assess the RT-qPCR data. The mean and standard derivation for efficiency, R^2^, slope, and y-intercept of RT-qPCR were 94.4 ± 2.2%, 0.996 ± 0.003, −3.466 ± 0.06 and 41.385 ± 1.09 and sample Cq variations did not diverge by more than 0.5 Cq. Four reference genes were evaluated in order to verify which of them would be the best to normalise the reactions. This analysis was performed by the RefFinder software (online version: https://www.heartcure.com.au/reffinder/ (accessed on 17 May 2017), which employs four diagnostic programs (BestKeeper, NormFinder, Genorm, and The comparative delta-Ct method). The most stable reference gene for samples analysed in the present study was *gapdh*.

**Figure 2:**
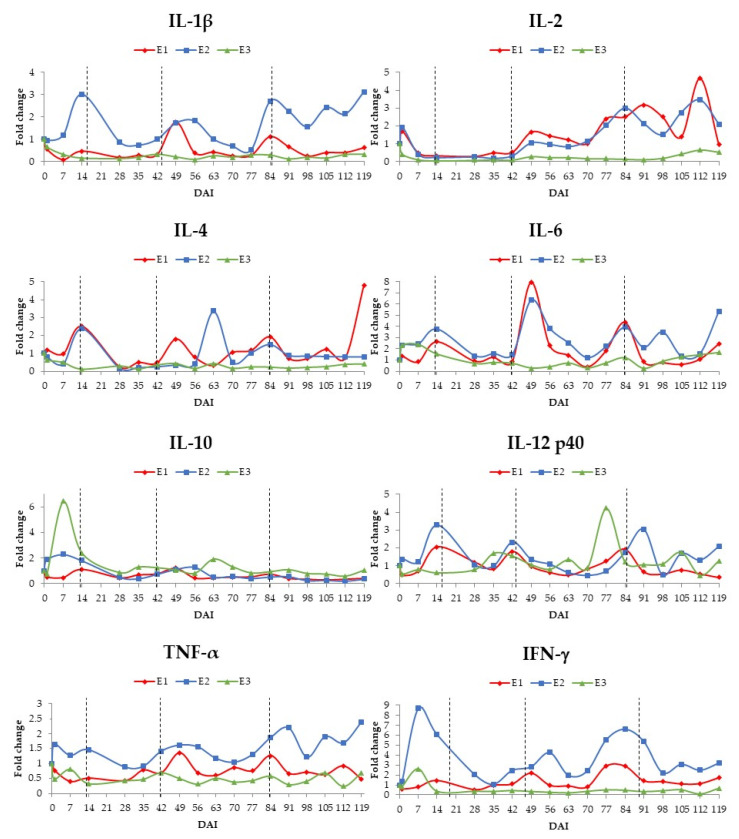
Relative quantification, by RT-qPCR, of IL-1β, IL-2, IL-4, IL-6, IL-10, IL-12 p40, TNFα, and IFNγ expression in peripheral blood cells from three *T. vivax* experimentally infected cows (E1 to 3). The expression of the target cytokines was normalised to *gapdh* as a reference gene.

The expression of each cytokine for each animal was compared to the expression at 0 DAI; the normalised Cq data for 0 DAI was set at one, and the fold-change in expression of each cytokine in following timepoints was calculated. The relative quantification for each cytokine is shown in Figure 2. When we compared these results with the parasitaemic curves quantified by qPCR (Figure 1), it was observed that both patent (A) and sub-patent (C) phases could be divided into two sub-phases. This division was based on the pattern of cytokine responses and changing in levels of parasitaemia. The early patent phase, A1 (from 1 to 14 DAI), saw the rise in parasitaemia as a consequence of inoculation; the late patent phase, A2 (from 14 to 42 DAI), was characterized by the apparent clearance of parasitaemia in the blood. The early sub-patent phase, C1 (from 42 to 84 DAI), was characterised by transient surges in parasitaemia, with fewer parasites than A1 and A2 intervals, or even aparasitaemic intervals. The late sub-patent phase, C2 (from 84 to 119 DAI), was characterised by the absence of *T. vivax* DNA, except for animal E3.

Each animal presented a unique and dynamic pattern of cytokine expression, with animals E1 and E2 showing a more similar pattern and animal E3 displaying a sustained reduction of expression for almost all cytokines during the infection (Figure 1 and Figure 2). At the individual animal level, two-fold changes in expression relative to 0 DAI at any point during a subphase was deemed as an increase (or decrease) in expression (Figure 1). If there was much fluctuation within the sub-phase, an average score was determined.

Relative to expression levels on 0 DAI, animal E1 presented in the A1 period a 2–3-fold increases in IL-4, IL-6, and IL-12 p40 expression, but 2–3-fold reductions in expression of IL-1β, IL-2, IL-10, and TNFα. During the A2 period, there were 2–3-fold reductions in expression of IL-1β, IL-2, and IL-4, with little change in expression for the other cytokines. The C1 period was distinctive for its overall elevation in cytokine expression with 5–6-fold increase in expression of IL-6, 2-fold increases of IL-2, IL-4, and IFNγ, and 2–3-fold reductions in expression of IL-1β and IL-10. During C2 period, animal E1 had a 2–4-fold elevation of IL-2, IL-4, and IL-6 expression, and no changes in TNFα and IFNγ, whereas all other cytokines underwent at least a 2-fold decrease in expression (Figure 1 and Figure 2).

As mentioned above, animal E2′s cytokine expression pattern over the course of infection showed similarities with that of animal E1. During the A1 period, animal E2 exhibited 3–7-fold increases in IL-1β, IL-12 p40, and IFNγ expression; 2–3-fold increases of IL-4, IL-6, and IL-10 expression, and a 2-fold reduction of IL-2 expression. In the A2 period, animal E2 had 2-fold elevation of IL-12 p40 and IFNγ expression, and 2–3-fold decreases in IL-2, IL-4, and IL-10 expression. These changes were followed in the C1 period by 2–6-fold increases in expression of IL-1β, IL-2, IL-6, TNFα, and IFNγ, and 2-fold reduction IL-10 expression. Finally, in the C2 period, animal E2 saw 2–4-fold rises of IL-1β, IL-2, IL-6, IL-12 p40, TNFα, and IFNγ expression and again a 2-fold reduction IL-10 expression (Figure 1 and Figure 2).

Animal E3 presented during the A1 period a 6-fold increase of IL-10 expression, 2-fold increases of IL-6 and IFNγ expression, alongside 5–10-fold reductions of IL-1β and expression and 2–3-fold fall of IL-2, IL-4, IL-12 p40, and TNFα expression. The A2 period saw the continued depression of transcription of most of the cytokines; expression of IL-1β and IL-2 showed 4–10-fold reductions, with 2–3-fold falls in IL-4, TNFα, and IFNγ expression. Only IL-12 p40 saw a modest increase (almost 2-fold). The C1 and C2 periods were also characterised by 3–10-fold depression of IL-1β, IL-2, IL-4, TNFα, and IFNγ expression, with the exceptions of a 3–4-fold elevation in IL-12 p40 expression during C1 (Figure 1 and Figure 2).

## 4. Discussion

Even though each animal presented a unique pattern of cytokine profiles, animals E1 and E2 presented a more similar response profile. They showed superior parasitaemia control, and a Th1 response during the patent phase was observed. These two animals generated an increase of Th2 cytokines during the C1 period, which may have enabled better control of the deleterious effects of a prolonged Th1 response [25]. Animal E3 that had least control of parasitaemia showed a decrease in expression for most of the cytokines evaluated. These findings suggest that a balance between the Th1 and Th2 cytokines profile, at least in peripheral blood cell expression, is beneficial for animal health and parasite control, and the absence of response may be deleterious to the animal.

Both pro-inflammatory (IFNγ, TNFα, and IL-12) and anti-inflammatory cytokines (IL-10) play an essential and similar role in trypanosomosis [14,26]. The VSG are the main inducing factors for TNFα production [27]. When TNFα is found in high concentrations, it is related to the cachexia typically observed in chronic infections by *T. vivax* [12], with anemia in *T. brucei* infections [28], and immunosuppression [29]. Naessens et al. [30] related the deficiency of this cytokine with the susceptibility of rats to *T. congolense* infection. Thus, TNFα production seems to be related to a parasitaemia control mechanism, since animal E2, in which an excellent response to *T. vivax* was observed, presented high expression of this cytokine during the sub-patent phase of the infection.

Even though animals from the present study showed no established pattern of IL-12 p40, the expression of this cytokine showed an increase in the patent phase and at the end of the C1 period. It is argued that IL-12 plays a role in the differentiation of T cells to Th1 [31]. Additionally, this cytokine stimulates the production of IFNγ by NK and T cells, increasing their cytotoxic activities [31,32]. Indeed, this is a more effective response against *Trypanosoma* sp. and *Leishmania* sp. protozoa [33]. IFNγ seems to be involved in parasitaemia control, thus contributing to the survival of the host in *T. b. brucei* [34], *T. b. rhodesiense* [35], and *T. cruzi* infections [36]. Even though animals from the present study presented different patterns of IFNγ expression, animals E1 and E2 showed increased expression of this cytokine. On the other hand, animal E3 predominantly presented a reduction of this cytokine, which seems to be related to inefficient parasite control.

IL-6 can act either as a proinflammatory or anti-inflammatory cytokine, with several implications on the pathophysiology of several diseases [26]. Herein, IL-6 expression was very similar for animals E1 and E2, since it was highly expressed especially in the sub-patent phase of infection. It is argued that IL-6 is involved in several biological activities, such as immune responses, haematopoiesis, and induction of acute-phase protein production by the liver [37]. Kato et al. [38] showed that an upregulation of IL-6 during the late stage of trypanosomosis is associated with a reduction in the severity of neurological involvement.

The IL-10 is a regulatory cytokine, which is secreted to control the exacerbation of inflammation [26]. Although IL-10 was expressed most at the A1 and C1 periods in the present study, no established pattern was observed. An increase in IL-10 inhibits the synthesis of Th1 cytokines (IFNγ, TNFβ, IL-1) and suppresses the action of NK cells and reactive oxygen species in activated macrophages [39]. It also acts by inhibiting the expression of IL-12, costimulators, and major histocompatibility complex (MHC) class II molecules [32]. Sternberg et al. [40] and Kato et al. [38] suggested that IL-10 is related to the protection of the central nervous system from pathological inflammatory processes.

IL-4 is a cytokine related to adaptive immunity [14]. The release of IL-4, a Th2 cytokine, is related to trypanotolerance in N’Dama cattle infected by *T. congolense*, which present high levels of IL-4 and low IL-6 [16]. In mice experimentally infected by *T. b. brucei*, IL-4 was related as a parasitaemia controller, playing a role on the immunoglobulin synthesis, but at the same time showing a toxic effect on the animals [41]. Animal E3, which showed no parasitaemia control, presented a reduction in the expression of this cytokine during the entire experimental period. On the other hand, animals E1 and E2 presented an increase in the expression of this cytokine during the C1 phase, thereby controlling parasitaemia.

IL-2 is a cytokine related to the proliferation, differentiation, and activation of T, B, and NK cells [32,42]. Reduction of this cytokine was observed in mice experimentally infected by *T. brucei* [43] and *T. congolense* [44], as well as in all animals of our experiment in the A2 phase of the infection and for cow E3 during the entire experiment. However, IL-2 expression was elevated for animals E1 and E2 in the sub-patent phase, probably as a response towards parasitaemia control.

## 5. Conclusions

The results found in the present study show a preliminary view of how the innate immune system works in cattle infected by *T. vivax*. Noteworthy, while IL-6 and IFNγ increased during most of the experimental period in animals that controlled the parasitaemia, cattle showing reduction of IL-1β, IL-2 and TNFα were not able to control the protozoan multiplication. Future studies should be performed with a more significant number of animals, and maybe for a shorter period, in order to establish a more robust pattern of cytokines profile and confirm the preliminary findings observed in the present study.

## Figures and Tables

**Table 1:** Oligonucleotide sequences and GeneBank access numbers for bovine IL-1β, IL-2, IL-4, IL-6, IL-10, IL-12 p40, TNFα, IFNγ, *gapdh*, β-actin, H3F3A, and YWHAZ genes.

Cytokine	Primers (5′-3′)	GenBank Access Number
IL-1β	F- CAAGGAGAGGAAAGAGACA R- TGAGAAGTGCTGATGTACCA	M37211
IL-2	F- TTTTACGTGCCCAAGGTTAA R- CGTTTACTGTTGCATCATCA	M12791
IL-4	F- CAAAGAACACAACTGAGAAG R- AGGTCTTTCAGCGTACTTGT	M77120
IL-6	F- TCCAGAACGAGTATGAGG R- CATCCGAATAGCTCTCAG	X57317
IL-10	F- TGCTGGATGACTTTAAGGG R- AGGGCAGAAAGCGATGACA	U00799
IL-12 p40	F- AACCTGCAACTGAGACCATT R- ATCCTTGTGGCATGTGACTT	U11815
IFNγ	F- ATAACCAGGTCATTCAAAGG R- ATTCTGACTTCTCTTCCGCT	M29867
TNFα	F- CCAGAGGGAAGAGCAGTCC R- GGCTACAACGTGGGCTACC	NM_173966
*gapdh*	F- TCGGAGTGAACGGATTCG R- ATCTCGCTCCTGGAAGATG	NM_001034034
β-actin	F- CGCACCACTGGCATTGTCAT R- TCCAAGGCGACGTAGCAGAG	K00622
H3F3A	F- GAGGTCTCTATACCATGGCTC R- GTACCAGGCCTGTAACGATG	NM_00101489
YWHAZ	F- GAAAGGGATTGTGGACCAG R- GGCTTCATCAAATGCTGTCT	NM_174814

## Data Availability

The data that support the findings of this study are available from the corresponding author (F.A.C) upon reasonable request.
